# Epidemiological, Morphometric, and Molecular Investigation of Cystic Echinococcosis in Camel and Cattle From Upper Egypt: Current Status and Zoonotic Implications

**DOI:** 10.3389/fvets.2021.750640

**Published:** 2021-10-04

**Authors:** Ahmed Gareh, Amira A. Saleh, Samar M. Moustafa, Amin Tahoun, Roua S. Baty, Refaat M. A. Khalifa, Ahmed K. Dyab, Doaa A. Yones, Mohsen I. Arafa, Amer R. Abdelaziz, Fatma A. El-Gohary, Ehab Kotb Elmahallawy

**Affiliations:** ^1^Department of Parasitology, Faculty of Veterinary Medicine, Aswan University, Aswan, Egypt; ^2^Department of Medical Parasitology, Faculty of Medicine, Zagazig University, Zagazig, Egypt; ^3^Department of Zoonoses, Faculty of Veterinary Medicine, Benha University, Benha, Egypt; ^4^Department of Animal Medicine, Faculty of Veterinary Medicine, Kafrelsheikh University, Kafrelsheikh, Egypt; ^5^Department of Biotechnology, College of Science, Taif University, Taif, Saudi Arabia; ^6^Department of Parasitology, Faculty of Medicine, Assiut University, Assiut, Egypt; ^7^Animal Health Research Institute, Assiut, Egypt; ^8^Department of Parasitology, Faculty of Veterinary Medicine, Sohag University, Sohag, Egypt; ^9^Department of Hygiene and Zoonoses, Faculty of Veterinary Medicine, Mansoura University, Mansoura, Egypt; ^10^Department of Zoonoses, Faculty of Veterinary Medicine, Sohag University, Sohag, Egypt

**Keywords:** molecular, morphometric, hydatid cyst, camel, cattle, Egypt, epidemiological

## Abstract

Cystic echinococcosis has been considered one of the major parasitic zoonoses which is associated with severe economic losses. The present study was undertaken to investigate the occurrence, organ distribution, cyst fertility, and viability of cystic echinococcosis in slaughtered camels and cattle from various abattoirs in Assiut Governorate, Egypt. The work also involved morphological, morphometric, and molecular identification of the parasite. The occurrence of hydatid cysts was investigated in total number of 100 lungs of camels and 574 liver and lungs of cattle admitted to three slaughterhouses at Assiut Governorate, Egypt. Moreover, several individual variable factors, including organ involvement, age, sex, and hydatid cyst characteristics, were studied to identify their possible association with the occurrence of the disease. Genomic DNA was extracted from the hydatid cysts, followed by molecular identification of the parasite through amplification of ribosomal DNA internal transcribed spacer (ITS) regions. Hydatid cysts were found in 6 camels (6%) out of 100 inspected camels, while 5 hydatid cysts (0.87%) were detected in a total number of 574 cattle examined. The parasite was detected exclusively in lungs of camels, while lungs were the main organ infected by the parasite in cattle and one hydatid cyst was found in the liver (0.17%). In camel, 66.7, 16.65, and 16.65%of detected cysts were fertile, sterile, and calcified, respectively, while in cattle, these percentages were 60, 20, and 20%, respectively. None of the studied variable factors were significantly associated with the occurrence of the disease in camels, with the exception that all cysts were found in the lung. Conversely, we found a significant association (*P* < 0.05) between the age and sex of the slaughtered cattle and the occurrence of hydatid cysts. In this respect, the rate of infection was higher in female cattle and those cattle more than 5 years (*P* < 0.05). The morphological, morphometric, and molecular studies confirmed the presence of the parasite. Taken together, our results concluded that camels and cattle play a potential role in maintaining the transmission cycle of this zoonotic parasite.

## Introduction

Cystic echinococcosis (CE) is a parasitic zoonotic disease with a worldwide distribution, particularly in developing countries (1–4). This disease is caused by a tapeworm of the species *Echinococcus granulosus* (EG) complex that has several distinct genotypes (5). The adult tapeworm inhabits the intestine of the definitive hosts, i.e., carnivores, who contract the infection through the consumption of the viscera of the intermediate hosts (3). The epidemiological profile of the disease includes human hosts and a wide range of wild and domestic hosts, including cattle, sheep, goats, and camels, which serve as intermediate hosts (3, 6). Carnivores then contaminate the environment, resulting in the spread of the infection, while human and other intermediate hosts contract the infection from fecal matter via oral routes and/or indirect contact with canines (3). In humans and other intermediate hosts, the larval stage of the parasite can reside and grow in the liver and lung, but rarely in other visceral organs (7). The pathogenesis of the disease and its clinical impact depend on the severity of the infection and the organ involved and the occasional rupture of hydatid cysts might lead to sudden death resulting from anaphylaxis, hemorrhage, and metastasis (8, 9). It is therefore not surprising to state that CE might represent a life-threatening disease in humans if left untreated, as it affects around one million people worldwide (10, 11). In addition, CE has been ranked as the second most significant helminthic disease associated with serious global economic losses, besides being a cosmopolitan disease (12–15). These huge economic losses result from the waste of animal protein because the edible organs are deemed unfit for human consumption (16, 17). According to the World Health Organization (WHO), echinococcosis results in around 19,300 deaths and 871,000 disability-adjusted life-years (18).

In accordance with its distribution, several previous studies revealed the endemicity of the disease in Egypt and neighboring countries of the Mediterranean basin and Middle East (19–30). The prevalence rates varied greatly among intermediate hosts of various species because of the influence of a variety of environmental factors and hygienic conditions (25), which is potentiated by the presence of infected stray dogs that are mostly used for guarding purposes or by the ease access of these dogs to slaughter houses (25, 29).

One of the most important strategies used for controlling the disease is accurate detection. The role of morphology criteria in the differentiation of the taxa of *E. granulosus* has been documented repeatedly (31–34). Several trials have been performed using light microscopy and, more recently, scanning electron microscopy (SEM) in detection of the parasite (34, 35). Light microscopy was reported as a valid method for identifying *E. granulosus* strains in several previous studies based on its larval hook morphology (33, 35). However, other studies have considered that its morphological identification based solely on ordinary microscopy is insufficient to differentiate the various strains of *E. granulosus* (34, 36–38). In this respect, SEM provides many advantages over light microscopy and facilitates the visualization and measurements of the large and small larval rostellar hooks, particularly in some countries where molecular studies cannot be performed (39, 40). Polymerase chain reaction (PCR) is a widely accepted molecular tool for epidemiological studies aimed at identifying and quantifying *Echinococcus* spp. in various tissues and body fluids, either in reservoirs or hosts (41–44). To our knowledge, *E. granulosus* is an assemblage of cryptic species that differ in morphology, host specificity, pathogenicity, and mitochondrial and nuclear genomes, making the taxonomy of this parasites is challenging issue (45). Several molecular approaches that can distinguish the genotypes of *E. granulosus* revealed that they are associated with distinct intermediate hosts, including sheep, goats, pigs, horses, cattle, camels, and cervids (5, 46, 47). Based on the most recent molecular phylogeny of the six nuclear genes and mitochondrial genomes, *E. granulosus sensu lato* comprises 5 species and 8 genotypes that represent intraspecific variants (48, 49). According to these analyses, G2 genotype has a variant of G3, while G6-G7 and G8-G10 genotypes are considered as distinct species. It seems that this great diversity has led to phylogenetic differences and affinities within the same genus. Therefore, it is not surprising to find that various hypotheses regarding the origin and geographic dispersal of the causative agents of CE have been reported (45).

Providing periodical updates on the available epidemiological data of the disease through field surveys performed for surveillance purposes, together with the investigation of the genotypes of the *E. granulosus* strains circulating in a given endemic area, may be crucial for the development of vaccines, diagnostic tests, and control strategies targeting hydatid disease (45, 50). Given the fact that limited information is available about the real contribution of camel and cattle in transmission of the disease in Upper Egypt, the present study was undertaken to investigate the occurrence, organ distribution, cyst fertility, and viability of CE in slaughtered camel and cattle from various abattoirs in Assiut Governorate, Egypt, followed by the morphological, morphometric, and molecular identification of the parasite.

## Materials and Methods

### Ethical Considerations

The study protocol was approved by the local guidance of Research, Publication and Ethics Committee of the Faculty of Veterinary Medicine, Kafrelsheikh University, Egypt, which complies with all relevant Egyptian legislations in publication and research. The ethical approval number is KFS-2015/1.

### Study Area and Sample Collection and Preparation

The present study was conducted to determine the occurrence of CE in camels (*N* = 100) and cattle (*N* = 574) slaughtered during the period of October 2015 to December 2017. The animals were admitted to different abattoirs (*N* = 3) in Assiut Governorate (Assiut, Bani-Adi, and Dairout abattoirs). Assiut Governorate is located in Upper Egypt (latitude, 27° 10′ 48.4824″ N; and longitude 31° 11′ 21.4188 ^″^W). The liver and lungs of camels and cattle were examined for the detection of hydatid cysts through visual inspection, palpation, and systematic incision of each lung (51). Any hydatid cysts obtained from the lungs and liver of each species were collected during the postmortem examination; then extracted, counted, and carefully removed during evisceration by dissection; and finally placed into sterile flasks (thermo flasks) and transported to the laboratory at the Department of Medical Parasitology, Faculty of Medicine, Assiut University, for the experimental work. The methodology included the morphological and microscopic identification of the hydatid cysts, together with sending samples of parasite material to the Molecular Biology Unit, Assiut University, Assiut, Egypt, for further examination and molecular analysis. The samples used for morphological and microscopic identification were preserved in 10% formalin and stored in closed containers at 4°C, while those used for molecular identification were kept in clean sterile bottles containing 70% ethanol (52).

### Morphological and Microscopic Examination of the Hydatid Cysts

The hydatid cysts were subjected to morphological examination to check their shape, size, viability, and condition according to a protocol described elsewhere (53, 54). The condition of the cysts was classified into three categories, as follows: fertile hydatid cysts, containing protoscoleces and/or daughter cysts; sterile hydatid cysts, full of fluid but without protoscoleces; and calcified hydatid cysts, with a tough thickened wall and absence of protoscoleces or fluid (53, 54). The hydatid fluid of each cyst was aspirated using 21 gauge needle. The collected fluid was centrifuged at 252 g for 5 min. The last drops of the sediment were then transferred to a slide, mounted with a glass cover slip, and observed under a microscope for the presence of protoscoleces, brood capsules, and taeniid hooks. When scolices could not be detected, the whole cyst was opened in a Petri dish, in which the fluid and germinal layer scrapings were examined for the presence of protoscoleces or brood capsules. Microscopic examination of the cyst fluid was performed to look for viable protoscoleces after dropping 0.1% eosin into the fluid. The specimens of hydatid cysts were also processed and prepared for SEM according to the protocol described elsewhere (40). Briefly, the hydatid fluid was aspirated from 3 hydatid cysts in cattle and 4 hydatid cysts in camels, then the parasite materials were prepared from the fluid by repeated centrifugations. This step was then followed by washing several times in phosphate buffer and fixation in mixture of 3% glutaraldehyde with 0.1 M phosphate buffer. The re-suspended samples were centrifuged twice at 112 g for 5 min and the resulting pellet was then resuspended in 1% osmium tetroxide prepared at room temperature. The morphometric analysis of the preparations was then done using SEM (Joel, JSM-5400LV Scanning Electron Microscope, Tokyo 1993, Japan) (40). The identified structures, including small and large hooks, were also examined using SEM for a different characteristics and aspects including length and the width of each hook and the guard angle, following to the protocols described elsewhere (39, 40, 55, 56).

### Preparation of Parasite Material for the Extraction of DNA

It is important to note that camel and cattle samples were processed separately using the same protocol. The processing of the cyst samples was carried out as described elsewhere (52). Briefly, the cyst wall was opened and the hydatid fluids were collected into marked test tubes, then centrifuged. The supernatant was discarded and the sediment (parasite material), which contains free scolices and brood capsules) was collected into clean sterile bottles containing 70% ethanol and stored at −20°C until use (52). After washing the samples with nucleic-acid-free water, to remove the ethanol, total genomic DNA was extracted from parasite material using the QIAamp DNA Mini Kit (QIAGEN, Germany), according to the manufacturer's protocol and as described elsewhere (50). The DNA was then stored in sterile DNAse- and RNAse-free microtubes and kept at −20°C.

### Molecular Identification (PCR)

Table 1 lists the primers used for the amplification of the DNA obtained from protoscoleces of hydatid cysts. The primers targeted the ribosomal DNA (rDNA) region spanning the internal transcribed spacers ITS-1 and ITS-2 regions which is validated for *E. granulosus* diagnosis (46, 57, 58). The PCR was designed for ITS1 and ITS2 regions and the amplification conditions were as per a protocol described elsewhere (5, 61), with slight modification. Briefly, the PCR mixture (25 μl) contained 12.5 μl of Master mix (Promega), 1 μl of forward primer (10 p/mol), 1 μl of reverse primer (10 p/mol), 1 μl of DNA (50 ng/μl of DNA template), and 9.5 μl of deionized distilled water. The amplification included an initial denaturation step of 5 min at 95°C; followed by 40 cycles of 1 min at 95°C, 30 s at 60°C, and 3 min at 72°C; and a final extension at 72°C for 10 min. Five microliter of the resultant amplified PCR products were analyzed in 1.6% (w/v) agarose gels in Tris-acetate-EDTA (TAE) buffer stained with ethidium bromide, transilluminated under ultraviolet light, and photographed. A known positive control comprising a reference strain was included [kindly provided by Professor Refaat Khalifa (Animal Health Research Institute, Assiut, Egypt)], while purified water was used as the negative control. Controls were processed in parallel with the experimental samples, to detect possible contamination.

**Table 1:** The PCR primers for rDNA region spanning the ITS region target genes.

**Gene**	**Primer type**	**Primer sequence**	**PCR product size (base pair)**	**References**
ITS region	ITS1-BD1Forward 4S (reverse)	5′GTCGTAACAAGGTTTCCGTA-3′ 5′TCTAGATGCGTTCGAATGTCGATG-3′	1,100 bp	(46, 57, 58)
	ITS2−3S- forward A28 - reverse	5′GGTACCGGTGGATCACTCGGCTCG3′ 5′GGGATCCTGGTTAGTTTCTTTTCCTCCGC-3′	750 bp	(57, 59, 60)

### Data Analysis

Data were collected, organized and then analyzed using SPSS, 23. Fisher's exact test was used to compare frequencies of presence of hydatid cyst in different organs in camels and cattle besides studying the potential explanatory individual variable factors associated with the occurrence of the disease. Furthermore, normality of quantitative parameters (length and width of hooks, handle, and blade of small hook and large hooks) were assessed using normal probability plots and the Kolmogorov-Smirnov test generated with the Proc *T*-test procedure of Statistical Analysis System (SAS^®^, version 9.2, SAS Institute, Cary, NC, USA) to study the statistical differences between cattle and camel. For all analyses, *P* ≤ 0.05 was defined as significant.

## Results

### Occurrence of Hydatid Cysts in Camel and Cattle Samples and the Potential Explanatory Individual Variable Factors

In the present study, hydatid cysts were detected in 6% of the examined lungs of camels. Table 2 summarizes the variable factors associated with the occurrence of the disease in camels. In accordance with organ distribution of hydatid cysts in camels, 6 hydatid cysts were detected in the lungs, whereas no cysts were found in the liver (*P* < 0.05). The remaining factors were not significantly associated with the occurrence of cysts. Regarding the age factor in camels, the rate of cysts was higher in camels older than 5 years of age than younger camels (<5 years of age). In this context, Table 2 shows that the occurrence of hydatid cysts in camels older than 5 years of age was 10% (4 out of 40 examined camels), whereas in younger camels (<5 years of age) this value was 3.33% (2 out of 60 examined camels). In accordance with sex, the occurrence of hydatid cysts in male camels was 6.7% (6 out of 90 examined camels) and no hydatid cysts were detected in females. Out of the 6 detected hydatid cysts, 4 (66.7 %) were fertile, 1 was sterile (16.65%), and another was calcified (16.65%). Furthermore, in camels, the current investigation showed that 75% (3 out of 4 examined cysts) of the fertile cysts were viable and 25% (1 out of 4 examined cysts) were non-viable. In addition, 3 hydatid cysts were found as single cysts in camels and another three animals experienced multiple hydatid cysts.

**Table 2:** Summarizes the data of the possible associations between the occurrence of hydatid cysts in camels and the potential explanatory individual variable factors of these detected cysts.

**Variable factor**		**Sample size**	**No. of positives**	**Positive rate/%**	**Statistical data (Fisher exact P. value)**
Organ	Lung	100	6	6	0.029*
	Liver	100	0	0	
Age	> 5 years	60	2	3.3	0.214
	<5 years	40	4	10	
Sex	Male	90	6	6.7	1
	Female	10	0	0	

*The symbol*

* *means that P < 0.05*.

In accordance with their occurrence in cattle, hydatid cysts were found in 0.87% of examined cattle. The potential explanatory individual variable factors associated with the occurrence of hydatid cysts in cattle are illustrated in Table 3. As shown in the table, the majority of hydatid cysts were detected in the lungs (0.7%), while one hydatid cyst was found in the liver (0.17%). The age and sex factors were the variables that were significantly associated with the occurrence of hydatid cysts (*P* < 0.05). The occurrence of hydatid cysts in female cattle older than 5 years of age was 2.6% (5 out of 175 examined cattle), while no hydatid cysts were detected in younger male cattle (<5 years of age). In addition, out of the 5 detected hydatid cysts, 3 (60%) were fertile, 1 was sterile (20%), and another was calcified (20%). Regarding the viability of fertile hydatid cysts, only 33.3% (1 out of 3 examined cysts) of the fertile cysts isolated from cattle were non-viable, while 66.7% (2 out of 3 examined cysts) were viable. In accordance with the number of cysts in cattle, 4 cattle had single cyst and only one animal exhibited multiple hydatid cysts.

**Table 3:** Summarizes the data of the possible associations between the occurrence of hydatid cysts in cattle and the potential explanatory individual variable factors of these detected cysts.

**Variable factor**		**Sample size**	**No. of positives**	**Positive rate/%**	**Statistical data (Fisher exact P. value)**
Organ	Lung	575	4	0.7	0.369
	Liver	575	1	0.17	
Age	> 5 years	400	0	0	0.003**
	< 5 years	175	5	2.6	
Sex	Male	400	0	0	0.003**
	Female	175	5	2.6	

*The symbol*

** *means that P < 0.01*.

### Molecular, Morphological, and Morphometric Identification of Hydatid Cysts

**Figure 1:**
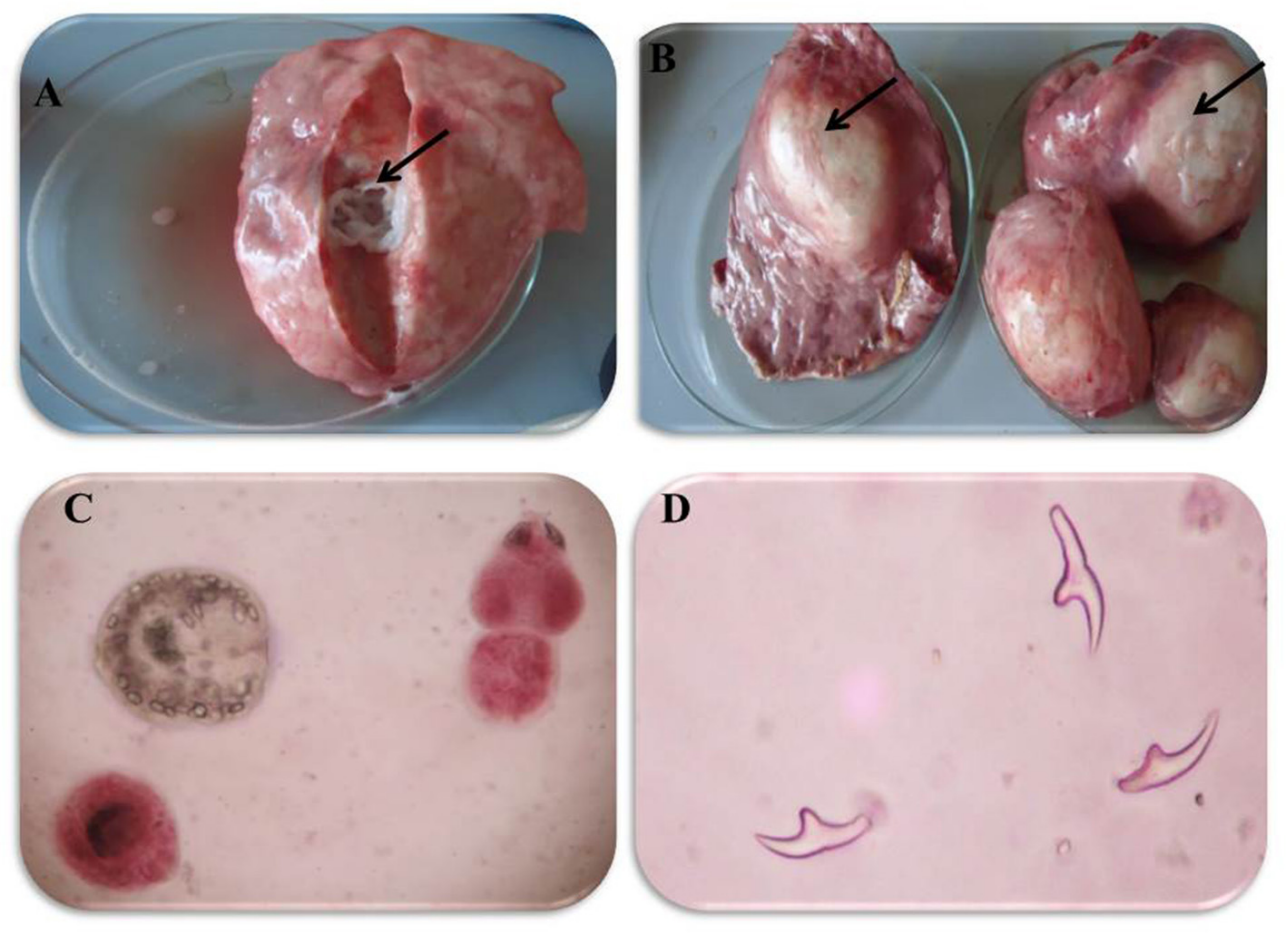
Morphological appearance of Hydatid cyst in the lung of camels. **(A)** Solitary sterile hydatid cysts. **(B)** Multiple hydatid cysts. **(C)** Multiple scolices in viable and non-viable fertile hydatid cyst, using 0.1% Eosin stain (X400) and **(D)** different-shaped and sized hooks in fertile viable-hydatid cysts.

**Figure 2:**
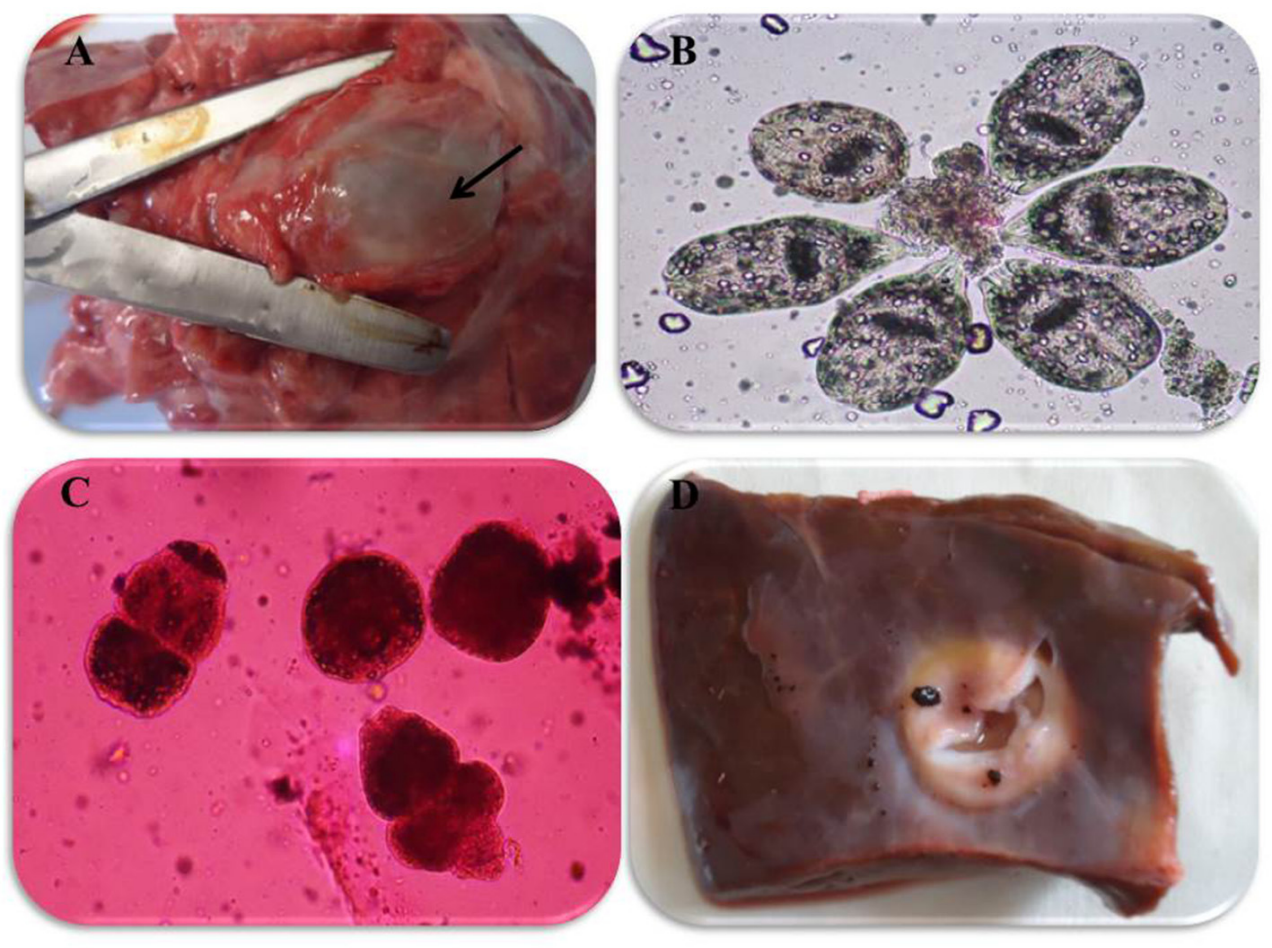
Morphological appearance of hydatid cyst in lung and liver of cattle. **(A)** Solitary viable fertile hydatid cysts in the lung of cattle. **(B)** Multiple scolices in viable fertile hydatid cyst in the lung of cattle using 0.1% eosin stain (X100). **(C)** Multiple scolices in non-viable fertile hydatid cyst in the lung of cattle using 0.1% eosin stain (X100) and **(D)** calcified hydatid cysts in the liver of cattle.

**Figure 3:**
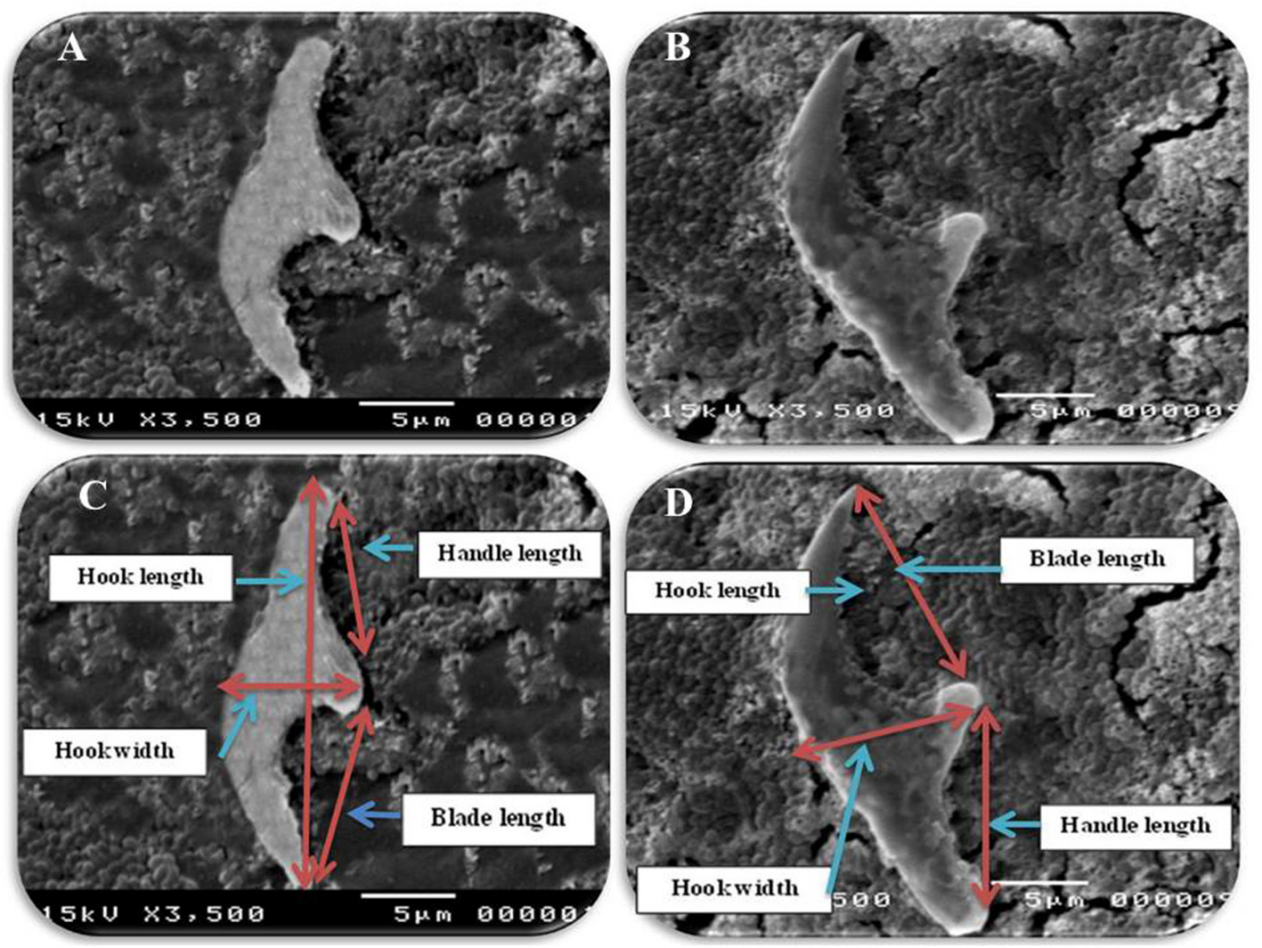
Morphometric appearance of small and large hook of hydatid cyst isolated from lung of camel using SEM. **(A)** The small hook of hydatid cyst. **(B)** The large hook of hydatid cyst. **(C)** Measurement the small hook of hydatid cyst; total hook length = 27 μm (arrow), total hook width = 9 μm (arrow), handle length = 9 μm (arrow) and blade length = 10 μm (arrow) and **(D)** measurement the large hook of hydatid cyst; Total hook length = 34 μm (arrow), total hook width = 12 μm (arrow), handle length = 14 μm (arrow) and blade length = 15 μm (arrow).

**Figure 4:**
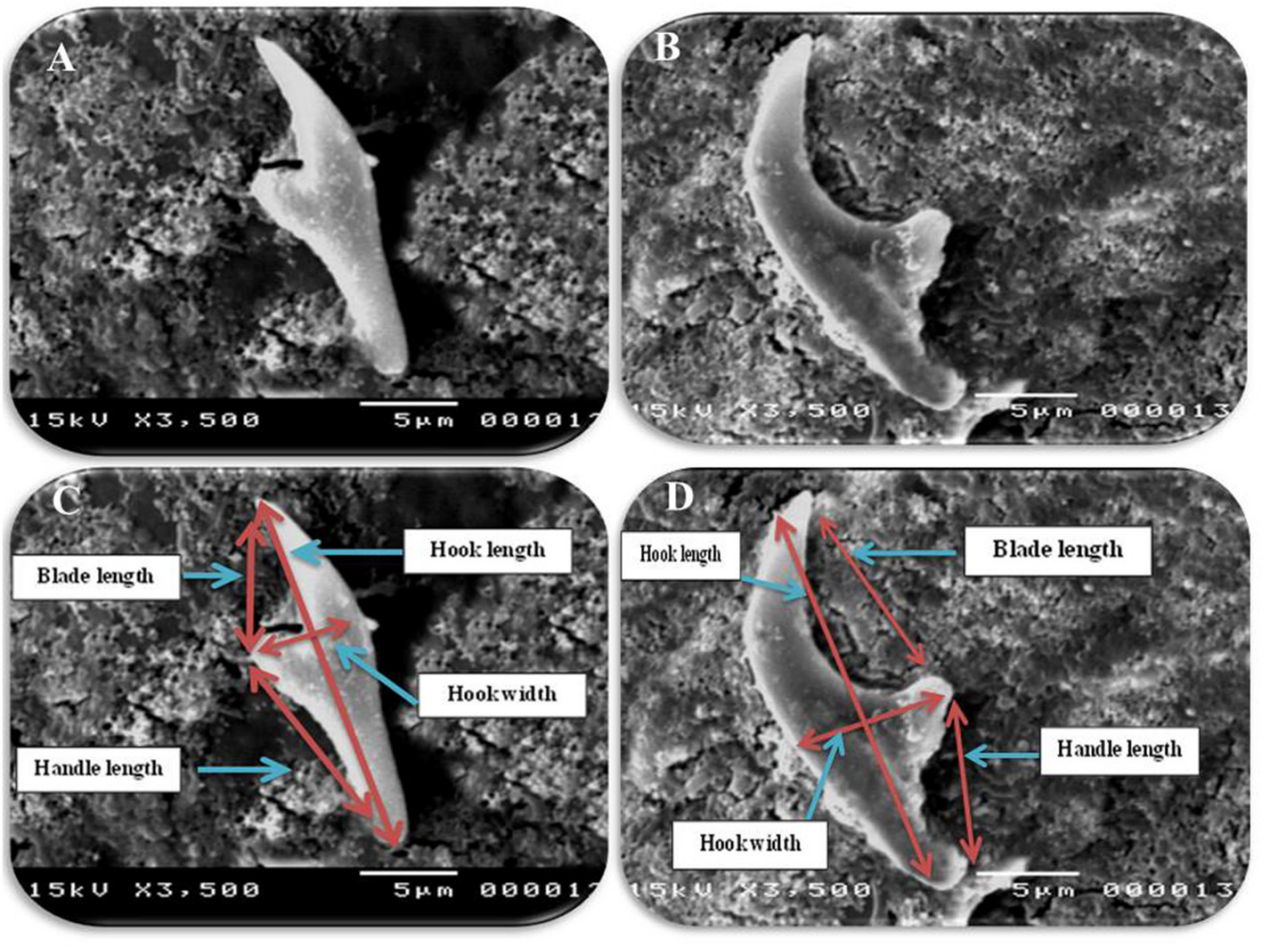
Morphometric appearance of small and large hook of hydatid cyst isolated from cattle lung using SEM. **(A)** The small hook of hydatid cyst isolated from cattle lung. **(B)** The large hook of hydatid cyst isolated from cattle lung. **(C)** Measurement the small hook of hydatid cyst isolated from cattle lung; Total hook length = 22 μm (arrow), total hook width = 6 μm (arrow), handle length = 10 μm (arrow) and blade length = 5 μm (arrow) and **(D)** measurement the large hook of hydatid cyst isolated from cattle lung; total hook length = 28 μm (arrow), total hook width = 8 μm (arrow), handle length = 8 μm (arrow) and blade length = 16 μm (arrow).

**Figure 5:**
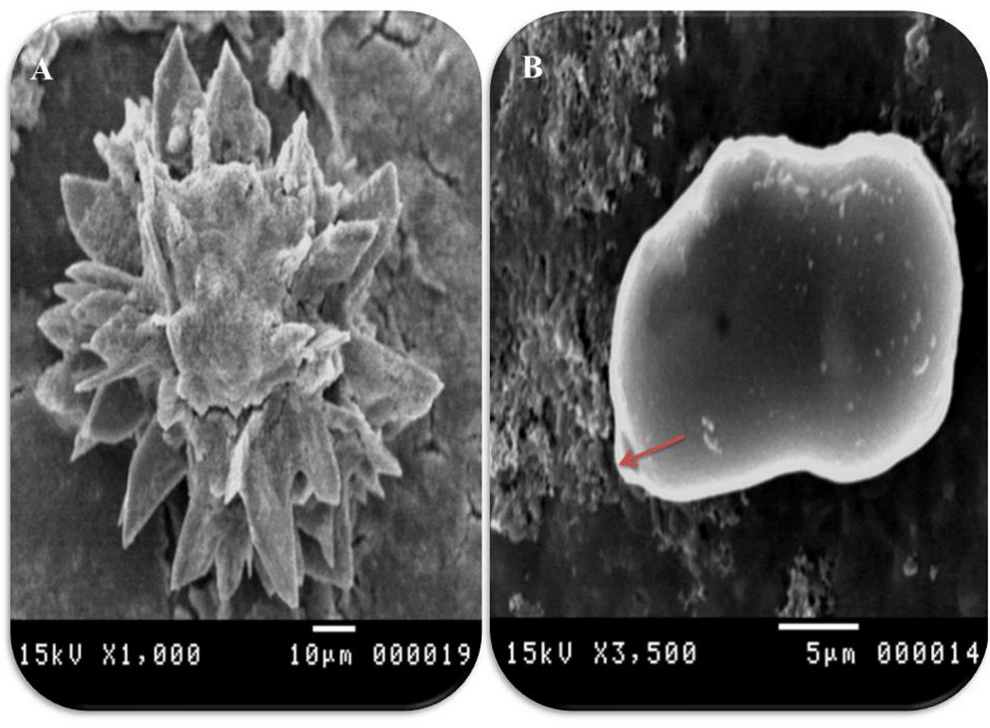
Morphometric appearance of hydatid cyst isolated from cattle lung using SEM. **(A)** SEM of rostellar hooks from protoscoleces of hydatid cyst, isolated from cattle showing alternating large and small hooks, and **(B)** SEM of brood capsules showing exterior outgrowth in hydatid cyst isolated from a cattle lung (arrow).

In accordance with the molecular identification of hydatid cysts (46, 57, 58, 62), the DNA fragments amplified from the rDNA extracted from hydatid cyst protoscoleces of the organs from infected camels and cattle were approximately 1,100 base pairs (bp) and 750 bp in length for ITS1 and ITS2, respectively (Supplementary Figures 1, 2). Figure 1 illustrates the gross morphological appearance of examples of the hydatid cysts detected in camels. Several instances of multiple scolices in viable and non-viable fertile hydatid cysts, and different-shaped and -sized hooks in fertile viable hydatid cysts in the lungs of camels were also observed (Figure 1). Similar to that observed in camels, the morphological and microscopic examinations of the hydatid cysts encountered in the lungs of cattle are shown in Figure 2, several instances of multiple scolices in viable and non-viable solitary fertile hydatid cysts, as well as different-shaped and -sized hooks in fertile viable hydatid cysts, were detected in the lungs of cattle (Figure 2). On the other hand, SEM visualization of the morphometric appearance of the small and large hooks of hydatid cysts obtained from the infected lung of camel is shown in Figure 3, while that of cattle are depicted in Figures 4, 5. The morphometric characteristics and measurements of the hooks of cattle and camel are shown in Table 4. As shown in Figures 3–5, morphologically, the small hooks of cattle lungs had a flatter concavity of the handle and much thinner blade with a pointed end, while the large hooks of cattle lungs were generally smaller than those of the camel lungs. Regarding their morphometry, the blade of small hooks of cattle lungs was narrow, the concavity between the handle and the guard was shallower, and the handle was longer and narrower than those of camel lungs. Moreover, the large hooks of cattle lungs were smaller in total length, width, and handle length, with a similar blade length. Collectively, Figures 3–5 depict the presence of slight but clear differences in the morphology and morphometry of the small and large hooks of cysts obtained from the lungs of camels and cattle.

**Table 4:** The morphometric measurements (μm) of total length, width, handle, and blade of large and small hooks hydatid cysts retrieved from lungs of cattle and camel [Values are means ± error of the mean (SEM)].

	**Cattle (mean± SD)**		**Camel (mean± SD)**	
	**Small hook**	**Large hook**	**Small hook**	**Large hook**
Length of hooks	22.32 ± 0.615^a^€	28.03 ± 0.97^¥^	34.05 ± 0.615^b^€	27.02 ± 0.44^¥^
Width of hooks	6.04 ± 0.16^b^€	8.03 ± 0.38^¥^	9.01 ± 0.34^a^€	12.05 ± 0.56^¥^
Handle length	10.07 ± 0.51€	8.04 ± 0.5^b¥^	9.06 ± 0.41€	14.02 ± 0.38^a¥^
Blade length	5.03 ± 0.34^b^€	16.02 ± 0.51^¥^	10.02 ± 0.63^a^€	15.02 ± 0.53^¥^

*Means within rows with different superscripts differ at P ≤ 0.05. Different letters, a and b, indicate statistical significance differences between small hook and large hook of cattle and camel. Different asterisks, ¥ and €, indicate statistical significant differences between values of small hook and large hook within the same species*.

## Discussion

The present study provides interesting data related to the occurrence of CE and to several potential explanatory individual variable factors associated with the occurrence of hydatid cysts in camels and cattle from slaughterhouses in Assiut Governorate, Egypt. The confirmation of the results was performed using a set of molecular identification tools and by studying the ultrastructure of the protoscoleces and hooks in the detected cysts via SEM. Given the fact, limited information are available about CE in upper Egypt, our study provides novel contribution about the occurrence of this disease and the real contribution of camels and cattle in maintenance the epidemiological foci of this disease of zoonotic importance in this area. As shown in our work, hydatid cysts were detected in 6% of examined lungs of camels. A previous survey of hydatid disease in camels and cattle in the same studied area reported a prevalence rate of 7.67% in camels, while no infection was reported in cattle (54). Another study reported a seroprevalence rate of 6% in camels harboring hydatid cysts (63). The frequency of CE observed in camels in the present work is similar to that detected previously by Dyab et al. (64) in Assiut Governorate (64) and to that reported by Lahmar et al. (65) in Tunisia (65). In contrast, higher occurrence rates of CE, of 9 and 24.15% were reported in camels from the same region of Egypt (66, 67). Moreover, Barghash et al. (26) reported higher rates of 18.7% in camels from Cairo, Dairut, Mallawy, and Kafr-ElShikh, Egypt (26). Another study of CE in various municipals abattoirs in Cairo, Giza, and Beni-Suef governorate, Egypt, reported a higher prevalence rate of 10.82% (29). Conversely, lower values of 2.35 and 5% have been reported in camels from Beni Suef and Upper Egypt, respectively (25, 68). In the present study, CE was only reported in 0.87% of examined cattle. The present rates of CE are lower than those of a previous study performed in cattle from Egypt, which reported an occurrence rate of 3.3% (69). Another study performed in Cairo reported that 18.4% of the examined cattle were infected by CE (70). In contrast, the occurrence rate obtained in the present study was higher than those reported in cattle slaughtered in abattoirs in Assiut, Mansoura, and other provinces of Upper Egypt, where hydatid cysts were noted in 0.4, 0.068, and 0.004% of the examined animals, respectively (25, 71, 72). The discrepancy in the occurrence of CE in camels and cattle in the present study vs. those reported in several previous studies may be attributed to several factors, such as hygienic practices during slaughter, sex and age of the slaughtered animals, the method of detection, the geographic location, and various climatic conditions (25, 45, 73, 74). The unhygienic disposal of condemned carcasses and infected organs, the ease of access stray dogs to slaughter houses, and the unauthorized slaughter are also relevant factors in the transmission of CE (29, 30, 74, 75).

In accordance with the studied potential variable factors, the statistical analysis revealed that the number and type of cysts, organ involved, and fertility and viability of hydatid cysts were not significantly associated with the occurrence of the of CE in camels or cattle. The present study also showed that CE were only detected in the lungs of camels, while cysts occurred mainly in the lungs of cattle and only one cyst was reported in the liver of cattle, which is consistent with several previous reports, mainly from Egypt (69, 70, 72). However, some previous reports revealed that the parasite exhibited single-organ involvement, although the involvement of two organs has also been recorded, which is apparently associated with the specific geographic regions and strain of the parasite (25, 76, 77). Regarding the age as variable factor, our study reveals that occurrence of hydatid cysts in aged camels at rate of 10% (4 out of 40 examined camels), whereas in younger male camels, this value was 3.33% (2 out of 60 examined camels). Furthermore, the present data illustrate the occurrence of hydatid cysts in aged cattle at a rate of 2.6% (5 infected out of 175 examined animals), while no infection was detected among young cattle (<5 years of age) (*N* = 400). The present findings agree with several previous studies performed either at the national (Egypt) or international level (70, 78, 79). This observation could be attributed to the fact that older animals are exposed to infection more than young ones and aged animals get slaughtered more than young ages, since their production (calves/milk) and working capacity decrease (80). Immunity represent an additional factor might involve this difference since older ages have weak immune system to combat the infection (81). Furthermore, the present study showed that female cattle were infected exclusively, at rate of 2.6% (5 infected out of 175 examined animals), while no infection was detected in the examined male cattle. This observation may be explained by the fact that female animals are not slaughtered at younger ages, as the owners mostly keep them for breeding, obtaining calves and for milk production; in contrast, male cattle are slaughtered at younger ages (70, 82, 83). The management practices might also contribute to this difference since males move far away for grazing; meanwhile females are usually kept homesteads, making females are more exposed to infection than males (84).

Considering the sex as potential variable factor, all detected hydatid cysts in camels were found in males and no cysts were detected in females. Meanwhile, all detected hydatid cysts in cattle were found in females. Reviewing the available literature, a previous study reported an infection rate of 16.89% in female and 13.55% in male cattle in Northwest Iran (85). This difference might be attributed to the low number of females analyzed in our study compared with males. In the present study, single cysts were detected in 80% of positive samples and multiple cysts were present in 20% of infected cattle, while single and multiple cysts were reported at an equal percentage of 50% in camels. Our results are consistent with those reported in Southern Italy, where 78.8% of the cysts were single entities (86). Regarding hydatid cyst viability in camels, 75% of the examined cysts were viable while 25% of the cysts were non-viable. Meanwhile, our data revealed that 66.7% of hydatid cysts detected in cattle were viable, while 33.3% of the examined fertile cysts were non-viable. Similar results were recorded in camels in Addis Ababa, where 66.6% of the detected hydatid cysts were viable (87). Another previous study performed in Southeastern Iran found that 57.14% of the hydatid cysts detected in camels were viable (88). Regarding the type of cyst, the current investigation showed that 4 of hydatid cysts (66.7 %) were fertile, 1 was sterile (16.65%), and another was calcified (16.65%). In addition, 75% of the fertile cysts in camels were viable and 25% were non-viable. Moreover, 3 hydatid cysts were found as single cysts in camels and another three animals experienced multiple hydatid cysts. On the other hand, our study found that 60% of hydatid cysts in cattle were fertile, 20% were sterile, and 20% were calcified. In addition, only 33.3% of the examined fertile cysts isolated from cattle were non-viable, while 66.7% were viable. Moreover, 4 cattle had single cyst and only one animal showed multiple hydatid cysts. Similar to the present results obtained in cattle, another study performed in Eastern Ethiopia found that 80% of the cysts were fertile, 17.3% were sterile, and 2.85% were calcified (79). Another study from Southern Italy revealed that 42.7% of cysts were sterile and 57.3% were calcified/caseous, while no fertile cysts were found (86). In this context, camelids and porcine are suitable hosts that frequently contain fertile cysts (45).

In accordance with the morphological and morphometric data (Table 4, Figures 3–5), the reported data indicated slight but clear differences in the morphology and statistical significant differences morphometric measurements of the small and large hooks obtained from the lungs of camels and cattle, which is consistent with the results of several previous studies (31–34, 39, 40). However, the limitations of the use of SEM, i.e., the need for infrastructure or financial constraints, favor the performance of molecular techniques vs. purchasing an SEM instrument (89).

Among other molecular targets, PCR was used to amplify the ITS region of ribosomal DNA as a genetic marker, which provides a simple and powerful tool for the accurate identification and differentiation of *Echinococcus* strains (90). In the present work, the size of the DNA fragment amplified for rDNA-ITS1 and rDNA-ITS2 was 1,100 and 750 bp, respectively, in both camel and cattle samples. Similar results were reported in several previous studies (57, 59, 62). A review of the available literature showed that CE is widespread in many countries where camels are raised, including Egypt (52, 91–94). This finding implies the widespread presence of CE across the area of Upper Egypt. Among others, recent molecular data suggested that the prevalence of infection of *E. canadensis G6* might be higher than previously described, and that this genotype exists as a complex of different strains that differ in a wide variety of criteria (92, 94, 95). Clearly, future genotypic characterization of the major strains circulating in the country using phylogenetic studies would provide interesting information about the genetic relatedness of the parasite, both at the regional and international levels.

## Conclusions and Recommendations

In summary, the results of this study demonstrated the occurrence of CE among camels and cattle in Upper Egypt. Moreover, our findings conclude that camels and cattle play a potential role in the maintenance of the zoonotic foci and the transmission cycle of the parasite in Egypt. Our results also suggest the possible spreading of this zoonotic disease to other provinces in Egypt, together with animal movements. Further research and epidemiological studies are recommended to explore the involvement of other intermediate hosts in Egypt and identify the species of *Echinococcus* species that are circulating throughout the country, which is important information for combating this zoonotic disease.

## Data Availability Statement

The original contributions presented in the study are included in the article/Supplementary Material, further inquiries can be directed to the corresponding author/s.

## Author Contributions

AG, RK, AD, DY, and MA involved in the conception of the research idea and methodology design, supervision, and performed data analysis and interpretation. AS, SM, AT, RB, FE-G, and EE participated in methodology, sampling, and data analysis. AG and EE drafted and prepared the manuscript for publication for publication and revision. RK, AD, DY, and MA contributed their scientific advice. All authors read and approved the final manuscript.

## Funding

This work was supported by the Taif University Researchers Supporting Program (Project number: TURSP-2020/269), Taif University, Saudi Arabia.

## Conflict of Interest

The authors declare that the research was conducted in the absence of any commercial or financial relationships that could be construed as a potential conflict of interest.

## Publisher's Note

All claims expressed in this article are solely those of the authors and do not necessarily represent those of their affiliated organizations, or those of the publisher, the editors and the reviewers. Any product that may be evaluated in this article, or claim that may be made by its manufacturer, is not guaranteed or endorsed by the publisher.
