# Supplementary material for: Epidemiological, Morphometric, and Molecular Investigation of Cystic Echinococcosis in Camel and Cattle From Upper Egypt: Current Status and Zoonotic Implications
Source: Front Vet Sci. 2021 Oct 4;8:750640. doi: 10.3389/fvets.2021.750640 (PMC8521178; doi:10.3389/fvets.2021.750640)
Supplement: Supplementary file 1 [file Table_1.DOCX]

Supplementary Material

## Supplementary Figures


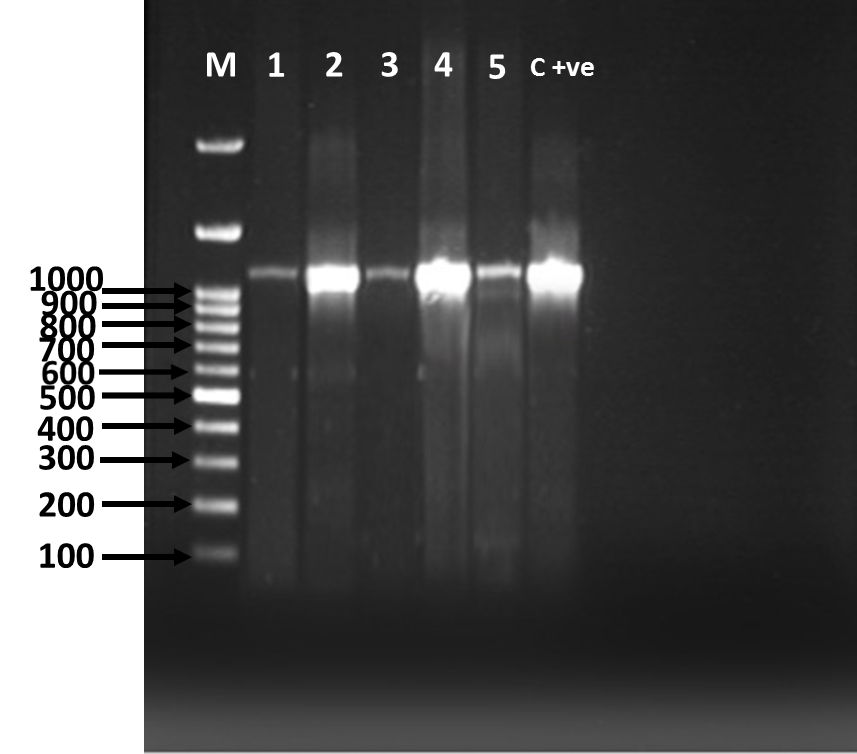


**Supplementary Figure 1.** Agarose gel electrophoresis of hydatid cyst isolated from lung of camel and cattle using 100-bp PLUS™ molecular standard size ladder (containing 12 linear sizing double-stranded DNA from 100 bp to 3,000 bp). The PCR targeted the amplification of internal transcribed spacer 1 (ITS1) of ribosomal gene with amplicon size of 1100 bp. Lanes 1-5 represent positive results for samples isolated from cattle (Lanes 1&2) and camel lung (Lanes 3-5), while lane 6 represent positive control.


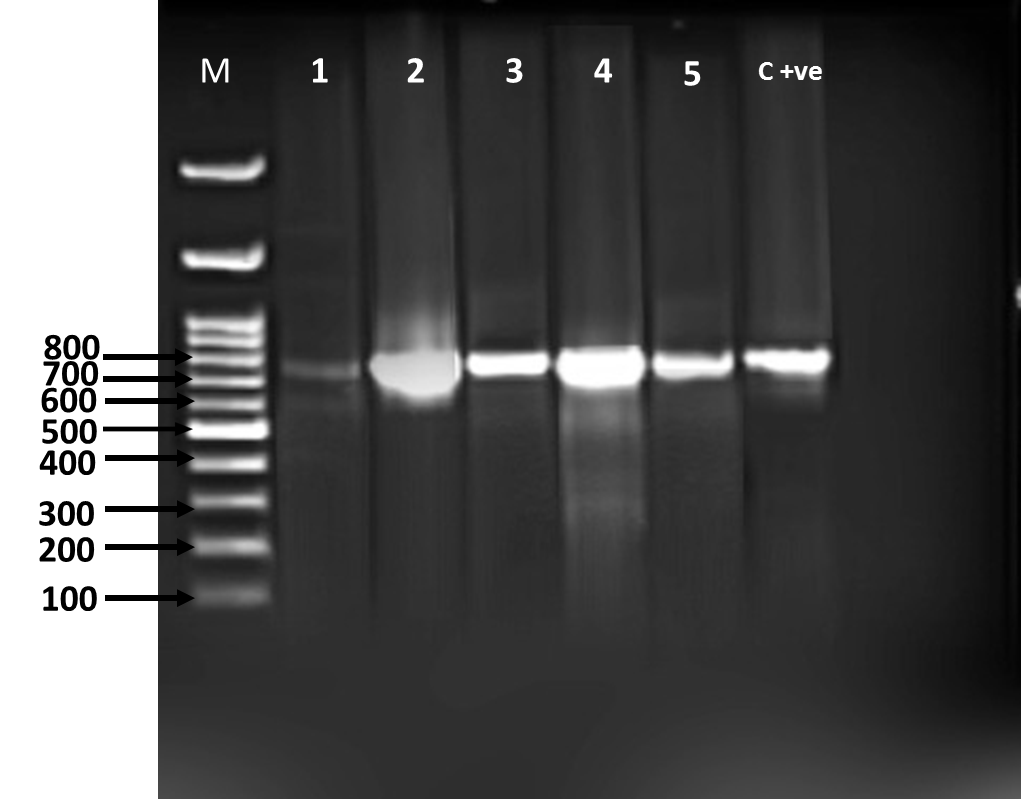


**Supplementary Figure 2.** Agarose gel electrophoresis of hydatid cyst isolated from lung of camel and cattle using 100-bp molecular PLUS™ molecular standard size ladder (containing 12 linear sizing double-stranded DNA from 100 bp to 3,000 bp). The PCR targeted the internal transcribed spacer (ITS2) of ribosomal gene of ribosomal gene with amplicon size of 750 bp. Lanes 1-5 represent positive results for samples isolated from cattle (Lanes 1&2) and camel’s (Lanes 3-5) lung, while lane 6 represent positive control.
